# Study Design of the Evaluation of Self‐Mediated Alternatives for Risk Testing Education and Return of Results ‐ E‐SMARTER: A randomized trial from the Alzheimer’s Prevention Initiative program

**DOI:** 10.1002/alz.089324

**Published:** 2025-01-09

**Authors:** Carolyn Langlois, Angela R. Bradbury, Elisabeth MCarty Wood, Kristin Harkins, Claire M Erickson, Emily A. Largent, Brian L Egleston, Eric M. Reiman, Joshua D Grill, J. Scott Roberts, Jason Karlawish, Jessica B. Langbaum

**Affiliations:** ^1^ Banner Alzheimer’s Institute, Phoenix, AZ USA; ^2^ University of Pennsylvania, Philadelphia, PA USA; ^3^ Department of Medical Ethics and Health Policy, University of Pennsylvania, Philadelphia, PA USA; ^4^ Fox Chase Cancer Center, Philadelphia, PA USA; ^5^ Institute for Memory Impairments and Neurological Disorders, University of California, Irvine, Irvine, CA USA; ^6^ University of Michigan School of Public Health, Ann Arbor, MI USA

## Abstract

**Background:**

Availability of amyloid modifying therapies will dramatically increase the need for disclosure of Alzheimer’s disease (AD) related genetic and/or biomarker test results. The 21st Century Cares Act requires the immediate return of most medical test results, including AD biomarkers. A shortage of genetic counselors and dementia specialists already exists, thus driving the need for scalable methods to responsibly communicate test results. The current study builds off our API Generation Program in which we demonstrated the safety and effectiveness of disclosing *APOE* and brain amyloid results via telemedicine to cognitively unimpaired adults.

**Method:**

The Evaluation of Self‐Mediated Alternatives for Risk Testing Education and Return of Results (E‐SMARTER) is a decentralized, randomized trial to evaluate self‐directed, scalable eHEALTH platforms for communicating *APOE*, brain amyloid, and plasma ptau217 test results, as well as to characterize the clinical impact of learning this information. E‐SMARTER will enroll approximately 600 cognitively normal adults aged 60‐80. Participants will be randomized to receive results either via telehealth videoconference or by eHealth platform. Participants randomized to the eHealth arm will be offered either the ADWebPortal or ADChatbot but can switch between platforms or request to speak with a healthcare provider during the sessions.

**Result:**

Study design and available participant demographics will be presented. Participants will complete online assessments evaluating their experience with the disclosure modalities as well as the impact of AD biomarker and genetic results disclosure. Participants complete assessments at baseline, prior to the initial *APOE*/amyloid PET disclosure session, and at approximately 0‐2 days, 6 weeks, 6 months, and 12 months following disclosure. pTau217 plasma levels will be assessed at 6 months after APOE/amyloid disclosure. PTau217 disclosure follow‐up assessments will be collected approximately 0‐2 days and 6 weeks after disclosure of pTau217 results. A final combined *APOE*/Amyloid and 6‐Month pTau217 follow‐up assessment will be collected approximately 12 months following the initial APOE/Amyloid disclosure.

**Conclusion:**

The E‐SMARTER study addresses the need for practical and scalable methods to responsibly communicate AD genetic and/or biomarker results. The E‐SMARTER eHEALTH platforms will be made available to support healthcare providers in returning genetic and/or biomarker results to individuals in clinical and research settings.

